# An open-source probabilistic record linkage process for records with family-level information: Simulation study and applied analysis

**DOI:** 10.1371/journal.pone.0291581

**Published:** 2023-10-20

**Authors:** John Prindle, Himal Suthar, Emily Putnam-Hornstein

**Affiliations:** 1 Suzanne Dworak-Peck School of Social Work, University of Southern California, Los Angeles, Los Angeles, California, United States America; 2 Keck School of Medicine, University of Southern California, Los Angeles, Los Angeles, California, United States America; 3 School of Social Work, University of North Carolina at Chapel Hill, Chapel Hill, North Carolina, United States America; King Khalid University, SAUDI ARABIA

## Abstract

Research with administrative records involves the challenge of limited information in any single data source to answer policy-related questions. Record linkage provides researchers with a tool to supplement administrative datasets with other information about the same people when identified in separate sources as matched pairs. Several solutions are available for undertaking record linkage, producing linkage keys for merging data sources for positively matched pairs of records. In the current manuscript, we demonstrate a new application of the Python RecordLinkage package to family-based record linkages with machine learning algorithms for probability scoring, which we call probabilistic record linkage for families (PRLF). First, a simulation of administrative records identifies PRLF accuracy with variations in match and data degradation percentages. Accuracy is largely influenced by degradation (e.g., missing data fields, mismatched values) compared to the percentage of simulated matches. Second, an application of data linkage is presented to compare regression model estimate performance across three record linkage solutions (PRLF, ChoiceMaker, and Link Plus). Our findings indicate that all three solutions, when optimized, provide similar results for researchers. Strengths of our process, such as the use of ensemble methods, to improve match accuracy are discussed. We then identify caveats of record linkage in the context of administrative data.

## Introduction

### Record linkage definition and motivation

Record linkage, also known as entity resolution, is the process of identifying records across unique datasets that capture information for the same entity. In the context of research, record linkage has been applied to merge administrative data to address research questions related to social sciences and public policy [1–3]. These studies leverage fields of interest (i.e., variables related to specific administrative processes) collected by public-sector and nonprofit agencies to assemble secondary datasets with a broader set of information than any single agency can provide.

Record linkage can be applied using deterministic (i.e., strictly identical matches and nonmatches) or probabilistic (i.e., calculating match probabilities between potential pairs) methods. Given the nature of administrative data, which may include missing, incomplete, or inaccurate values for fields (e.g., misspelled names or incorrect birth dates), there is an advantage to implementing probabilistic linkage methods, and studies have shown data simulated to mimic real linkage parameters are more accurately linked with probabilistic methods than deterministic methods [4]. Additionally, applying probabilistic linkage methods to large datasets, for which it is often not feasible to conduct human reviews, can output quality linkage results without a cumbersome and resource-intensive manual review process [5].

### Probabilistic record linkage process

The key steps in probabilistic record linkage are: (1) standardizing and cleaning data; (2) blocking; (3) generating a comparison feature matrix; and (4) assigning match probability. The standardization and cleaning step ensures that all columns are comparable between the two datasets in the linkage by performing transformations such as removing special characters from fields, capitalizing letters in name and address fields, and setting invalid data to blanks (e.g., Social Security number values of 00-00-0000 or first names values of “Baby Boy”). The blocking step reduces all possible record pairs between the two datasets in the linkage to only the most likely pairs, which can be implemented via hard rules (e.g., only pairs that match on first letter of last name), fuzzy matching (e.g., only pairs whose birth years are within 2 years of each other), or both. The next step performs pairwise comparisons between the blocked pairs, exporting the results in the form of a Boolean matrix, where a row represents a pair to be compared and a column represents the comparison (e.g., first names are an exact match). Based on the results of this matrix, a match probability is assigned to each row via human review or machine algorithm and a final decision is made on whether the pair is a match. Further reading on probabilistic record linkage can be found in Christen [6], Sayers et al. [7], and Harron et al. [8].

### Current landscape of probabilistic record linkage

Link Plus, developed by the Centers for Disease Control and Prevention [9], is a manual review solution designed to detect duplicates in cancer registries and link these registries to external sources. It assigns a match score to potential match pairs based on similarity of personally identifiable information values, presents these values of potential pairs in descending order of score, and allows end users to determine a match threshold after clerically reviewing the scored pairs. This solution does not apply a standard cleaning process to the datasets to be linked (i.e., linkage results may differ across users if different cleaning mechanisms are implemented), demands a high time investment (due to the manual review process), has an upper limit of 4.5 million to 4.8 million rows for the first dataset in the linkage (dependent on the hardware specifications of machine; the second dataset does not have a row limit), only runs on Windows, and outputs results that are highly subjective to the end user determining the match threshold score. When done correctly, however, it produces highly accurate results because most positive matches have been reviewed manually [2]. It is also relatively easy to implement for users with less technical knowledge.

ChoiceMaker [10] is a fully automated solution that only requires users to specify the datasets they wish to link and the training data from which to generate scoring algorithms. Due to its low computing and user input requirements, it is scalable to large datasets and can handle a high throughput of linkages with little manual involvement. Further, due to the automated nature of the scoring process (i.e., performed by a model), results of linkages are consistent across runs, with accuracy in line with Link Plus. Still, this solution does not include a standard cleaning process or guidelines, is compatible only with specific computing environments, requires a significant time commitment in specifying a training dataset, and offers only limited user customization options outside of training data procurement and determining a match probability cutoff.

Other solutions such as Link King [11], Dedupe [12], Match*Pro [13], and splink [14], although not benchmarked in the current paper, have different combinations of the strengths and shortcomings relative to the previously mentioned solutions. The RecordLinkage package in R was considered due to its open-source nature but ultimately not reviewed due to its inability to be easily translated to run parallel processing [15]. No solutions reviewed adequately address the shortcomings of Link Plus and ChoiceMaker while also duplicating their strengths.

### Probabilistic record linkage for families (PRLF) and how it differs

#### Definition

PRLF was developed to allow end users more control over the linkage task while automating as many steps as possible. The solution is an automated, Python-based, end-to-end linkage pipeline built on the open-source RecordLinkage package (see https://recordlinkage.readthedocs.io/). PRLF performs data cleaning, blocking, feature extraction, model training, and match probability assignment while also generating optimized models for scoring based on various algorithms of interest and training data supplied. All components of PRLF are accessible via a single install, and although it has default settings that allow users to perform linkages “out of the box,” users easily can control parameters at each step of the linkage process to fit their needs with minimal editing of Python code. Further, PRLF is agnostic to system software and hardware and can be run on Windows, Mac OS, and Linux machines of varying hardware capabilities. A detailed process flow and available user customizations can be found in S1 Text and S1 Fig.

#### Key features

PRLF is automated by design, and the Python architecture allows for throughput and scalability comparable to ChoiceMaker with a low level of human intervention during the linkage process. The high degree of customizability over each step provides the potential for a high degree of match accuracy in line with Link Plus. The Python architecture obviates the need for third-party support, instead empowering the user to edit the open-source Python code to tune the tool to a specific linkage. The user arguments pertaining to hardware performance allow PRLF to be portable enough to run on even the most basic hardware specifications. Finally, PRLF is expandable to allow for numerous machine learning algoirthms (e.g., regularized regression, random forests, XGBoost) to estimate match probabilities, which allows for the creation of ensembles of probability scores for further scrutiny to identify matched pairs. Thus, PRLF builds on the combined strengths of current linkage solutions while addressing their gaps. A comparison of PRLF with other linkage solutions is displayed in Table 1.

**Table 1:** Features of probabilistic linkage solutions available for record linkage applications.

Solution	Link Plus	ChoiceMaker	Link King	RecordLinkage (R)	PRLF
**Operating system**	Windows	Windows	Windows, Linux	Windows, Mac, Linux	Windows, Mac, Linux
**Architecture**	NA	Java	SAS	R/C	Python
**Customizability options**					
Fields from each dataset to compare to one another	Yes	Yes	Yes	Yes	Yes
Blocking fields and algorithms	Yes	No	Partial	Fields only	Yes
Comparison features generated	No	With support	No	Yes	Yes
Batch size for memory optimization	No	No	No	No	Yes
Serial or parallel operation	No	No	No	No	Yes
Training data	NA	Yes	Yes	Yes	Yes
Scoring algorithm types and hyperparameters	NA	No	Partial	Partial	Yes
Match probability or score threshold	Yes	Yes	Yes	Yes	Yes
Handling of multiple matches	No	No	Yes	No	No
**Capacity limitations**	4.8M per dataset	Dependent on hardware configuration	99.9M per dataset	Dependent on hardware configuration	Dependent on hardware configuration
**Supported file types**	Text-delimited file	Database	SAS dataset	Text-delimited file or database	Comma-separated values file or database

### Study aims

In the current study, we compared several known probabilistic record linkage solutions and PRLF, a novel record linkage solution designed to provide researchers with greater control over characteristics dictating linkage performance. We conducted comparisons using two methods: simulation and applied analysis.

#### Simulation analysis

First, we ran PRLF to link sets of simulated data with known parameters to provide insight into the performance of the linkage solution. Our simulation analysis was guided by several questions: (1) How well does blocking correctly document true matches? (2) Are blocking accuracy and block size associated with dataset size, percentage of overlap, and percentage of rows with typos introduced? (3) What is the proportion of matches identified in the blocked pairs and how does it compare to the total match pairs? (4) What is the proportion of false positives?

#### Applied analysis

Next, we compared the linkage results of PRLF to other linkage solutions using real-world administrative data. For our applied analysis, we sought to address the following questions: (1) Does PRLF, with trained algorithms, perform similarly to other linkage solutions? (2) Does varying the PRLF algorithm threshold for identifying a match pair affect analyses based on linkages? (3) Is it possible to establish recommended thresholds that researchers can use as a starting point and modify based on desired accuracy?

## Methods

### Simulation analysis

#### Data

The data created through simulation were designed to mimic data observed in records from California (i.e., administrative data for health and human services agencies serving residents of the state) [16]. Simulated datasets were created in pairs with the purpose of treating each pair as two distinct datasets to be linked (see S2 Text for details on the data creation process, along with the Python script used to generate the simulated datasets). Paired datasets had the same three parameters: (a) number of unique individuals in the dataset (i.e., row count); (b) percentage of overlap between the two datasets (i.e., percentage of identical rows); and (c) percentage of rows in each dataset with some form of information error introduced. Each dataset contained the following fields: source ID, first name, middle name, last name, birthdate, street address, city, state, and ZIP code. The source ID field served as a unique identifier for each row in a dataset and was created using a random number generator, ensuring no duplicates in a dataset. The fields of first name, middle name, last name, street address, and city were generated using the Python package Faker version 13.15.1 (see https://faker.readthedocs.io/en/master/index.html). The birthdate field was created using a random number generator, ensuring all birthdays were in 2016. The state field was set to “California” for all individuals, and the ZIP code field was populated using random selection (with replacement) from all valid California ZIP codes (performed once per row). The dataset sizes included 10,000, 50,000, and 100,000 rows. The row percentage overlap parameter values were 1%, 3%, 5%, 10%, 20%, 40%, and 50%. The percentage error parameter values were 0%, 2%, 5%, 10%, 15%, 20%, 30%, and 40%. In total, 168 pairs of datasets were created, one pair for each parameter.

#### Linkage

All simulated pairs underwent the “out of the box” PRLF processes (S1 Fig) for data cleaning and standardization, blocking, and generation of a comparison feature matrix. The first step identified which fields were to be used for linkage, standardized variable names used in the matching process, and allowable values in records to be compared between sources. Potential match pairs were then selected through a preliminary blocking stage that identified similar first or last names through a term frequency–inverse document frequency (TF-IDF) algorithm [17] with parameters of 4 n-gram length, top 3 matches of names matching at least 80%, and an additional constraint of matching birth year and month between records. Features were then extracted from this filtered set of potential match pairs using comparison functions across fields of unique and nonunique identifiers (see S3 Text and S1 Table for a sample of comparison functions). Using the resulting Boolean matrix from the previous step, record pairs were scored using an extreme gradient boosting (XGBoost) algorithm optimized through a grid search on prototypical training data [18]. XGBoost was selected for examining simulation and applied data linkages to provide a simplified interpretation of implementation of probabilistic data linkage in the context of PRLF. Further comparisons of alternative algorithms and ensemble methods may be produced. The grid search hyperparameters included learning rate (0.01, 0.10, 0.25), max depth (5, 10, 15), and number of estimators (250, 500, 1,000, 2,000). All potential match pairs after blocking in each dataset pair were assigned match probabilities using the same model.

#### Simulation analysis

Comparison of model performance was done with two main simulation parameters, overlap between dataset pairs and percentage of rows with administrative errors. Several metrics for exploring the resulting linkages from simulated data were presented from a typical confusion matrix. That is, pairs identified as matches (positives) and nonmatches (negatives) through model scoring were identified as true or false based on the data simulation procedure. We calculated the sensitivity, specificity, precision, and F1 score for each condition in the simulation grid. We additionally calculated metrics of blocking performance (proportion of true match pairs in blocked data and proportion of true matches in all blocked data).

### Applied data analysis

#### Data

Data from the California Department of Public Health Vital Statistics was obtained for the birth cohort year 2016 (*N* = 487,853). These data were linked to child protection service (CPS) records from the California Department of Social Services from 2016 (*N* = 5.8 million unique individuals). Identifiers for both datasets included child names (first, middle, last); date of birth; parent or guardian names (first, middle, last) and dates of birth; and complete addresses (street, city, state, ZIP code). These identifiers were used in three linkage solutions: Link Plus, ChoiceMaker, and PRLF. Link Plus and ChoiceMaker have been used for previous analyses with these data [19, 20], and PRLF was compared to these accepted linkage solutions. Data available to the research team were stripped of direct identifiers and anonymized prior to analysis when accessed in January 2023. Analyses were approved by the California Health and Human Services Agency’s Committee for the Protection of Human Subjects and met criteria for a waiver of informed consent. Work with these data was also reviewed by the Vital Statistics Advisory Committee and data security protocols were approved by a university institutional review board.

#### Linkage parameters

Link Plus allows users to identify blocking variables and subsequent to the blocking procedure, use various field comparisons to identify potential matched pairs between data sources. ChoiceMaker similarly has a blocking step, with more sophisticated field comparison functions that are used as features for a machine learning model to output a match probability per potential record pair. Although Link Plus requires manual review of potential record pairs without prior model training, ChoiceMaker allows the user to set a threshold for which a pair of records will be called a match (set at 80% match probability for the model used on the current data). For PRLF, we used the combination of these approaches with birth year, birth month, first name, middle name, and last name meeting a similarity threshold based on TF-IDF to establish a set of blocked record pairs for which to generate comparison features. Pairs were placed into a block if their first names or last names were in the top 3 matches as determined by TF-IDF, with a match probability cutoff of 80% using n-grams of length 4 (excluding leading and trailing spaces). Of these pairs, only those that matched on exact birth month and birth year continued to feature extraction. The extracted features of these blocked pairs were then used to determine a match probability using the XGBoost algorithm. The parameterization for this model is based on a grid search outlined in the simulation data linkage solution. We compared results of the PRLF linkage with varied thresholds for establishing a positive match between data sources to those of the established solutions.

#### CPS outcome variable

Referral for abuse or neglect to CPS (the first stage of contact with CPS) was identified by a positive linkage between the birth cohort and a CPS maltreatment record with a referral within 3 years of the birthdate of birth (yes or no). A 3-year window of observation was used based on data available at the time of data linkage (i.e., maltreatment referrals through 2019).

#### Birth cohort characteristic variables

Birth records offered covariates as a measure of model accuracy using various linkage solutions. Child sex (male or female), low birthweight (yes or no) and variables related to the mother: age (< 20, 20–25, 26–30, >30), race and ethnicity (White, Black, Hispanic, Asian or Pacific Islander, other), non-U.S.-born (yes or no), and education (high school graduate; yes or no). Variables related to the household were also coded: public insurance (yes or no), first trimester prenatal care (yes or no), established paternity (yes or no), and WIC (supplemental services provided for low-income women, infants, and children with nutrition risk; yes or no). Later sections show the distribution of these variables in the subset of children for each linkage solution.

#### Applied analysis

An initial bivariate comparison of linkage tools tested the equality of group distributions on the outcome (CPS referral) and model covariates from the birth records using chi-square tests. Then, a generalized linear model with a Poisson distribution with a log link function and robust standard errors was used to show the multivariable relationship of the covariates with CPS referral likelihood [21]. To test the effect of match threshold selection, a range of thresholds above and below a relatively conservative 80% threshold was established (40%–95%) and the tests previously outlined were performed (chi-square tests and generalized linear modeling analysis). Proportions for varied thresholds were tested for trends to provide a starting point for establishing a threshold with linkages performed with the PRLF solution.

## Results

### Simulation analysis

Simulations were run for the grid of 168 linkages specified in the methodology. Table 2 shows an iteration of the results for a simulation run with 50,000 rows, with varying row overlap between datasets and varying errors introduced in a subset of rows. The results of the XGBoost algorithm are presented with a prediction probability threshold for match set at 80%. The table shows seven metrics of model performance: (a) accuracy; (b) sensitivity; (c) specificity; (d) precision; (e) F1 score; (f) proportion of true matches in blocks; and (g) sensitivity adjusted to include true matches not in block. As depicted in Panel A, two trends occurred: an increased proportion of true matches between datasets was indicative of increased accuracy, and these gains were seen across increasing levels of jitter or noise added to records. Accuracy improved as errors in records increased for low levels of record overlap (0.732 to 0.820 at 0.01 overlap), but the opposite was true for higher levels of record overlap (0.940 to 0.910 at 0.50 overlap). Panel B shows that as errors increased, sensitivity decreased, but there was no interaction with the amount of overlap between datasets. Panel C exhibits how specificity increased with the proportion of rows with introduced error (0.727 to 0.818 at 0.01 overlap), with no interaction with percentage of overlap. On the other hand, in Panel D, we see precision increasing with percentage of overlap (0.063 to 0.928 at 0.00 row errors) and no interaction with proportion of rows with introduced error. Panel E documents the F1 score decreasing with proportion of introduced error and increasing with data overlap. These effects are the harmonic mean of precision and sensitivity, which researchers may value over the combined metric when determining model appropriateness for specific use cases. In Panel F, we observe that the proportion of true matches identified by blocking decreased with an increase in row errors, in line with the proportion of row errors introduced. No interaction was found with the proportion of overlap between the datasets. Finally, in Panel G, the proportion of true matches in the blocked record pairs increased as the proportion of overlapping records between datasets increased.

**Table 2:** Fit statistics for scored XGBoost models with 50,000 rows per dataset.

Overlap	Proportion of rows with errors							
	0.00	0.03	0.05	0.10	0.15	0.20	0.30	0.40
A. Accuracy (higher is better)								
0.01	0.732	0.734	0.745	0.756	0.770	0.778	0.801	0.820
0.03	0.743	0.747	0.756	0.766	0.778	0.786	0.806	0.827
0.05	0.754	0.758	0.764	0.771	0.783	0.793	0.809	0.829
0.10	0.780	0.784	0.789	0.804	0.806	0.808	0.825	0.845
0.20	0.829	0.823	0.829	0.835	0.837	0.844	0.860	0.859
0.40	0.910	0.906	0.907	0.902	0.902	0.899	0.899	0.893
0.50	0.940	0.937	0.936	0.930	0.925	0.923	0.914	0.910
B. Sensitivity (higher is better)								
0.01	1.000	1.000	0.998	0.998	0.988	0.992	0.982	0.969
0.03	1.000	1.000	0.999	0.995	0.984	0.982	0.980	0.954
0.05	1.000	0.998	0.996	0.996	0.993	0.986	0.974	0.961
0.10	1.000	0.998	0.998	0.994	0.989	0.984	0.977	0.968
0.20	1.000	0.999	0.997	0.996	0.988	0.984	0.975	0.961
0.40	1.000	0.999	0.997	0.992	0.989	0.985	0.975	0.959
0.50	1.000	0.999	0.997	0.994	0.990	0.984	0.972	0.958
C. Specificity (higher is better)								
0.01	0.727	0.729	0.741	0.752	0.766	0.775	0.798	0.818
0.03	0.728	0.733	0.743	0.754	0.768	0.777	0.798	0.822
0.05	0.729	0.735	0.743	0.751	0.765	0.777	0.797	0.820
0.10	0.732	0.738	0.746	0.766	0.773	0.777	0.801	0.829
0.20	0.735	0.729	0.743	0.758	0.768	0.786	0.820	0.829
0.40	0.739	0.740	0.757	0.765	0.786	0.796	0.826	0.842
0.50	0.743	0.744	0.762	0.776	0.788	0.810	0.832	0.856
D. Precision (higher is better)								
0.01	0.063	0.062	0.063	0.064	0.063	0.062	0.062	0.061
0.03	0.173	0.174	0.174	0.173	0.171	0.172	0.168	0.167
0.05	0.268	0.268	0.266	0.265	0.266	0.266	0.257	0.249
0.10	0.448	0.451	0.448	0.456	0.445	0.431	0.433	0.426
0.20	0.673	0.662	0.665	0.662	0.658	0.654	0.654	0.626
0.40	0.878	0.873	0.873	0.864	0.859	0.852	0.843	0.825
0.50	0.928	0.924	0.923	0.915	0.908	0.905	0.892	0.882
E. F1 score (higher is better)								
0.01	0.118	0.116	0.119	0.119	0.119	0.116	0.116	0.114
0.03	0.296	0.297	0.297	0.295	0.291	0.293	0.287	0.284
0.05	0.423	0.423	0.420	0.419	0.420	0.419	0.407	0.396
0.10	0.619	0.622	0.618	0.625	0.614	0.599	0.600	0.592
0.20	0.804	0.796	0.798	0.795	0.790	0.786	0.783	0.758
0.40	0.935	0.932	0.931	0.924	0.920	0.914	0.904	0.887
0.50	0.963	0.960	0.959	0.953	0.947	0.943	0.930	0.918
F. Matches in block (higher is better)								
0.01	0.994	0.960	0.934	0.890	0.826	0.764	0.672	0.588
0.03	0.997	0.971	0.932	0.874	0.813	0.781	0.664	0.586
0.05	0.998	0.972	0.935	0.887	0.831	0.780	0.672	0.567
0.10	0.997	0.977	0.932	0.883	0.824	0.766	0.680	0.572
0.20	0.997	0.971	0.936	0.879	0.832	0.773	0.663	0.576
0.40	0.998	0.976	0.936	0.881	0.821	0.772	0.673	0.580
0.50	0.998	0.974	0.942	0.881	0.824	0.772	0.669	0.580
G. Adjusted sensitivity (lower is better)								
0.01	0.000	0.000	0.000	0.000	0.000	0.000	0.000	0.000
0.03	0.001	0.001	0.001	0.001	0.001	0.001	0.001	0.001
0.05	0.002	0.002	0.002	0.002	0.002	0.002	0.002	0.002
0.10	0.004	0.004	0.004	0.004	0.004	0.003	0.004	0.003
0.20	0.007	0.007	0.007	0.007	0.007	0.008	0.007	0.006
0.40	0.014	0.014	0.014	0.014	0.014	0.015	0.014	0.014
0.50	0.017	0.016	0.017	0.018	0.018	0.018	0.017	0.017

Notes. Accuracy describes the model’s ability to predict true positives and true negatives. Sensitivity describes the model’s ability to identify true positives. Specificity describes the model’s ability to identify true negatives. Precision describes the model’s ability to identify false positives. F1 is an aggregate score combining and weighing sensitivity and precision equally. Matches scored after blocking represent the proportion of true matches included in blocking. Adjusted sensitivity includes the true matches not included in blocking when calculating.

Results were replicated with 10,000 and 100,000 rows per dataset pair and provided comparable results, as presented in S4 Text and S2 and S3 Tables.

### Applied analysis

#### Training accuracy

Training data consisted of 5,771 record pairs of both known matches and nonmatches. These pairs included various prototypes of matches that would provide notably discriminable data for identifying high probability of a positive match (and conversely, pairs providing information about how to discern nonmatches). Parameters for each algorithm were selected through a grid search process, and parameters that maximized accuracy were selected. Hyperparameters are specific to the algorithm selected. PRLF includes the capability to train additional classifiers available in SciKit Learn with model-specific grid search parameters and selects the hyperparameters producing the best fit. Examples of other trainable models include logistic regression, random forests, XGBoost, and artificial neural networks. The XGBoost algorithm with match probability threshold set at 80% produced a solution that identified 93.8% of true matches, with 1.4% of nonmatches mislabeled a match. This is similar in performance to ChoiceMaker (match probability threshold of 80%, 94.7% of true matches identified, and 2.0% of nonmatches mislabeled as a match) and neural networks (match probability threshold of 75%, 96.8% of matches correctly identified, and 1.7% of nonmatches mislabeled as a match). Logistic regression (match probability threshold of 80%, 89.9% of true matches identified, and 4.3% of nonmatches mislabeled as a match) and random forests (match probability threshold of 40%, 95.0% of true matches identified, and 4.3% of nonmatches mislabeled a match) exhibited inflated levels of false positives compared to the other algorithms. As a result of these comparisons, the applied data example compared the performance of PRLF with XGBoost to Link Plus and ChoiceMaker.

#### Applied linkage accuracy

Records from birth records and CPS records were linked using the three linkage tools: Link Plus, ChoiceMaker, and PRLF with XGBoost. The manual review of Link Plus record pairs yielded a linkage rate of 12.0% of the total birth record population (*n* = 58,377) to CPS before age 3 years, and ChoiceMaker provided a 12.2% (*n* = 59,685) linkage rate with a 80% threshold for match probability as suggested by ChoiceMaker and in line with prior research using current linkage solutions. These two methods overlapped with 97.18% of Link Plus pairs and 95.05% of ChoiceMaker pairs. PRLF using XGBoost identified 12.1% (*n* = 59,102) birth records as a match to a CPS record. When compared to the other solutions, 95.6% of PRLF pairs matched with Link Plus record pairs and 97.2% PRLF pairs matched with ChoiceMaker record pairs. As shown in Table 3, when the threshold for identifying a match pair varied (40%–95%), the proportion of overlap trended upward as the threshold for identifying match pairs increased (94.8%–96.1%). That is, as the threshold for a positive match increased, only more certain matches remained and the two datasets fell more in line with each other. The tradeoff with increasing the PRLF threshold is a lower percentage of Link Plus pairs matched (99.8% to 97.8% over the range of PRLF match percentage thresholds). A similar trend in solution comparisons is shown between ChoiceMaker and Link Plus in S4 Table, where higher match percentage thresholds yielded more certainty in overlapping pairs with a lower total of match pairs.

**Table 3:** PRLF match pair overlap with Link Plus match pairs.

Threshold	Pairs matched by PRLF	True pairs matched by PRLF	Pairs matched by PRLF only	Pairs matched by Link Plus only	True PRFL pairs in all pairs matched by PRLF (%)	True PRLF pairs in all pairs matched by Link Plus (%)
0.40	59,925	56,832	3,093	123	94.80	99.80
0.45	59,826	56,822	3,004	133	95.00	99.80
0.50	59,725	56,794	2,931	161	95.10	99.70
0.55	59,597	56,719	2,878	236	95.20	99.60
0.60	59,490	56,646	2,844	309	95.20	99.50
0.65	59,404	56,611	2,793	344	95.30	99.40
0.70	59,285	56,566	2,719	389	95.40	99.30
0.75	59,191	56,526	2,665	429	95.50	99.20
**0.80**	**59,102**	**56,474**	**2,628**	**481**	**95.60**	**99.20**
0.85	58,927	56,395	2,532	560	95.70	99.00
0.90	58,715	56,283	2,432	672	95.90	98.80
0.95	57,961	55,708	2,253	1,247	96.10	97.80

Notes. Thresholds varied for PRLF results only; 56,474 match pairs were identified by both PRLF and Link Plus and 56,955 total match pairs were identified in the Link Plus linkage solution. The 80% threshold is highlighted in bold to emphasize the selected threshold used in this applied data analysis.

#### Applied linkage analysis

The generalized linear model was fit to each linked data source: birth to CPS using PRLF, Link Plus, and ChoiceMaker. The birth cohort proportions and subsequent CPS subpopulations in the cohort are shown for each linkage method (see Table 3). These proportions in each linkage groups (i.e., children identified as having a CPS report before age 3) are similar across each linkage method, indicating that the small discrepancy of match pairs between linkage methods did not significantly alter the underlying analytic characteristics. Further, Table 4 compares the cohort distribution of birth record variables to the distributions in each linkage subpopulation. Significant differences occurred between the subpopulations on all demographic variable distributions and that of the overall cohort. All comparisons in Linkage subpopulations showed no significant distributional differences except maternal nativity ($\chi_{df=2}^{2}=13.2,p=.001$).

**Table 4:** Demographic characteristics of the 2016 California birth cohort.

			CPS linked birth records					
	Full Birth Cohort		PRLF		Link Plus		ChoiceMaker	
Variable	*n*	%	*n*	%	*n*	%	*n*	%
Male	249,915	51.2	26,784	52.0	26,288	51.9	27,053	52.0
Mother’s race and ethnicity								
White	137,356	28.2	12,312	23.9	12,321	24.3	12,410	24.3
Black	26,502	5.4	6,581	12.8	6,566	13.0	6,697	13.0
Hispanic	228,622	46.9	28,323	55.0	27,515	54.3	28,625	54.3
Asian or Pacific Islander	65,044	13.3	2,003	3.9	2,037	4.0	2,046	4.0
Other	30,329	6.2	2,249	4.4	2,207	4.4	2,291	4.4
Maternal nativity†	185,913	38.1	11,381	22.1	10,859	21.4	11,635	21.4
Mother’s age (years)								
< 20	21,474	4.4	5,057	9.8	4,978	9.8	5,085	9.8
20–25	213,055	43.7	28,445	55.3	28,057	55.4	28,764	55.4
26–30	231,559	47.5	16,493	32.0	16,183	32.0	16,713	32.0
30+	21,718	4.5	1,456	2.8	1,421	2.8	1,497	2.8
High school graduate	394,826	80.9	35,168	68.3	34,838	68.8	35,436	68.8
Low birthweight	33,320	6.8	5,020	9.8	4,939	9.8	5,143	9.8
Prenatal care								
1–3 months	401,594	82.3	36,231	70.4	35,668	70.4	36,510	70.4
3–6 months	59,251	12.1	9,523	18.5	9,400	18.6	9,650	18.6
7–9 months or missing	27,008	5.5	5,714	11.1	5,578	11.0	5,909	11.0
Public insurance	222,865	45.7	39,233	76.2	38,449	75.9	39,827	75.9
WIC	223,733	45.9	36,374	70.7	35,694	70.5	36,848	70.5
Paternity established	456,308	93.5	41,722	81.1	41,269	81.5	42,169	81.5

Notes. WIC = Women, Infants, and Children status for program supports. Chi-square tests for distribution differences were significant between overall cohort and each linkage across all demographic variables. †A significant difference was found for maternal nativity between linkages (*p* = .001).

**Fig 1:**
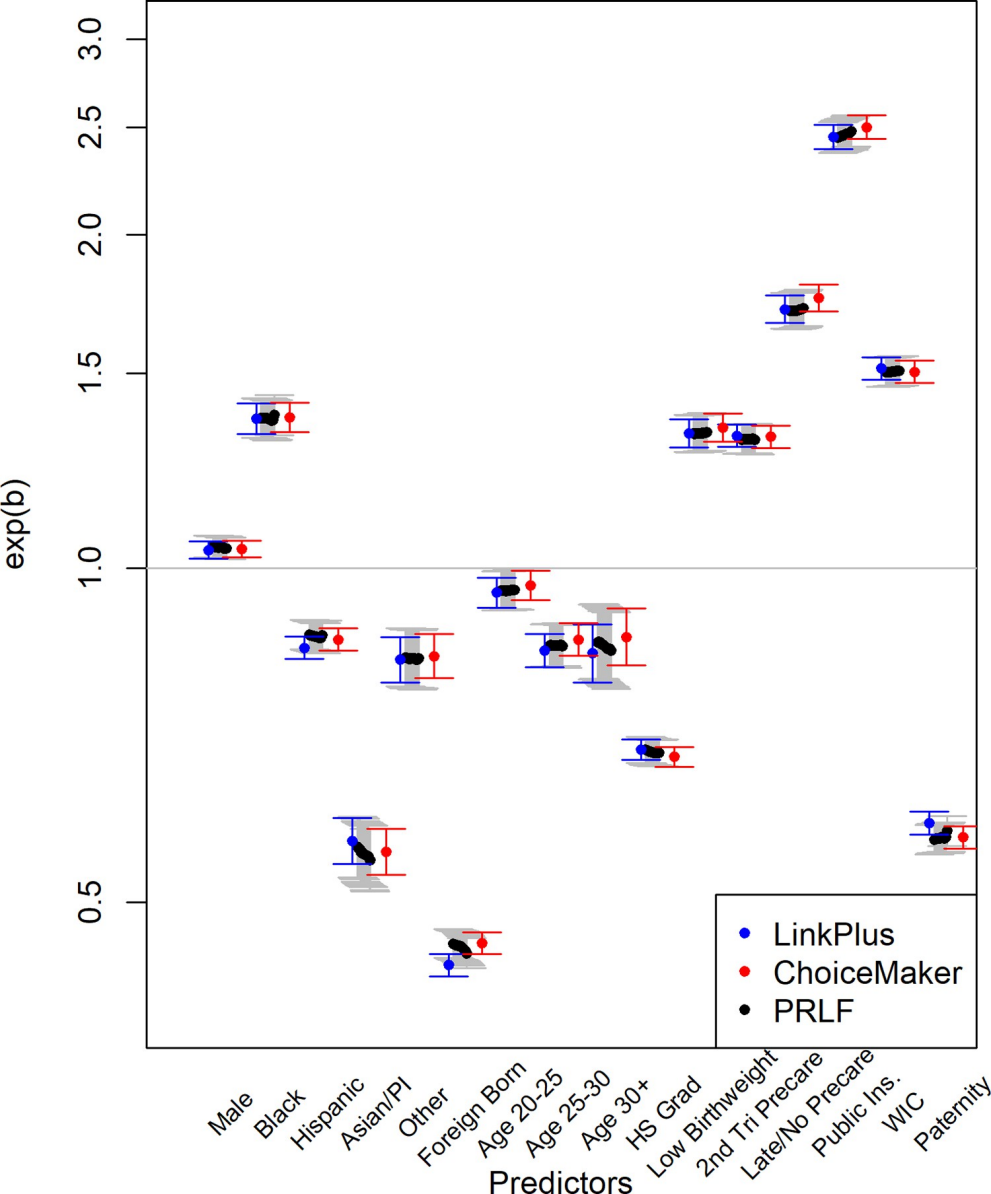
Comparison of PRLF with varied match thresholds to Link Plus and ChoiceMaker model estimates with 95% confidence intervals. Estimated parameters for each predictor is shown with a solid dot and confidence intervals are represented by error bars. Blue identifies Link Plus parameter estimates and confidence intervals and red identifies ChoiceMaker estimates and confidence intervals. Black dots and grey confidence intervals show the estimates for the XGBoost algorithm of PRLF when the threshold for a pair identified as a match varied (0.40–0.95) from left to right. Values above exp(b) = 1 indicate an increase in likelihood of child welfare referral, whereas values below exp(b) = 1 indicate a decrease in the likelihood of child welfare referral.

Table 5 displays the estimated parameters for each predictor with referral by age 3 as the outcome. These estimates and 99% confidence intervals show agreement between solutions where different linkages identified the subpopulation of children with a CPS referral. These findings largely mirror those previously found for similar linkages with previous years of data [22–24]. That is, several factors were associated with increased risk of CPS referral (e.g., low birthweight, public insurance, later prenatal care), whereas other factors were associated with reduced risk (e.g., paternity established and high school graduation). Fig 1 shows how varying the threshold of acceptable match probabilities affected model prediction (with Link Plus and ChoiceMaker parameter estimates to show relative differences). In the range of probability thresholds examined (40%–95%), there does not appear to be significant bias in parameter estimates using PRLF with an XGBoost algorithm to link data. The confidence intervals of the established linkage solutions encompass the range of values the XGBoost algorithm estimated across all birth characteristics.

**Table 5:** Generalized linear modeling parameter estimates of birth characteristics predicting CPS referral by age 3.

	PRLF with XGBoost			Link Plus			ChoiceMaker		
Parameter	RR	99% CI		RR	99% CI		RR	99% CI	
Male	1.04	1.02	1.07	1.04	1.02	1.06	1.04	1.02	1.07
Black	1.36	1.31	1.42	1.36	1.31	1.42	1.37	1.31	1.43
Hispanic	0.87	0.84	0.89	0.85	0.82	0.87	0.86	0.84	0.89
Asian or Pacific Islander	0.55	0.52	0.59	0.57	0.53	0.60	0.56	0.52	0.59
Other	0.83	0.78	0.88	0.83	0.78	0.88	0.83	0.78	0.89
Foreign born	0.45	0.44	0.47	0.44	0.43	0.45	0.46	0.45	0.47
Mother’s age									
20–25	0.96	0.92	1.00	0.95	0.91	0.99	0.96	0.93	1.00
26–30	0.85	0.81	0.89	0.84	0.81	0.88	0.86	0.83	0.90
30+	0.85	0.78	0.92	0.84	0.77	0.91	0.87	0.80	0.94
High school graduate	0.68	0.66	0.70	0.69	0.67	0.71	0.68	0.66	0.69
Low birthweight	1.32	1.27	1.38	1.32	1.27	1.38	1.34	1.29	1.39
Prenatal care									
3–6 months	1.31	1.27	1.35	1.32	1.28	1.36	1.31	1.28	1.35
7–9 months or missing	1.71	1.65	1.78	1.71	1.65	1.78	1.75	1.69	1.82
Public insurance	2.47	2.39	2.55	2.45	2.37	2.53	2.50	2.42	2.58
WIC	1.51	1.46	1.55	1.51	1.47	1.56	1.50	1.46	1.55
Paternity established	0.57	0.55	0.59	0.59	0.57	0.61	0.57	0.55	0.59

Notes. PRLF with XGBoost results are based on a probability threshold of 80%. RR = risk ratio. CI = confidence interval.

## Discussion

Based on the simulation analysis, we found that the PRLF linkage solution performed comparably to other open-source and widely adopted linkage solutions, including ChoiceMaker and Link Plus. Linking data involves several stages in which researchers may have different requirements for the process, including cleaning functions to apply to available personally identifiable information fields to be compared; type and strictness of blocking algorithms; which algorithms are implemented in scoring comparison features of potential pairs; and at what probability to accept a match (i.e., how high the bar is set for matching people between sources). The PRLF solution allows researchers to adjust these settings and compare results across various linkage assumptions. The applied analysis identified a typical research use case of PRLF—the linkage of a population-based vital records dataset with an administrative dataset and analysis of program interaction for a birth cohort.

The simulation analysis identified the various metrics affected by row errors and dataset overlap. Many performance metrics improved as the theoretical overlap between the dataset pairs increased, in line with expectations for well-defined linkage solutions. Accuracy and precision showed the largest improvements, with other metrics stable for a specified proportion of row errors. That is, as the proportion of overlapping records increased between two datasets, the proportion of false positives fell while holding the match threshold constant.

Alternatively, the introduction of row errors reduced the ability of blocking to successfully retain true matches between datasets. This is shown in the proportion of matches identified in the blocked data (Table 2, Panel F). Although the prior metrics showed negligible effects from this degraded signal, it necessary to note that data linkage relies on accurate records from sources. Random error across datasets indicates a robust linkage solution can use the correct information across fields to obtain linkages in the range of: *Proportion Blocked* = 1−*mean_row error_*. Two data sources with relatively clean records (i.e., free from error) would produce a blocked set with nearly all true matched record pairs.

Researchers may adjust the threshold for an acceptable match pair between datasets (or similarly, implement several algorithms for estimating the probability of match pairs). The applied analysis presented here shows how threshold selection, examined over a large range (40%–95%), can be tested in the context of PRLF if other linkage tools have been used. Similarly, the development of PRLF allows for multiple algorithms to be deployed simultaneously. Machine learning encompasses a wide variety of methods for classification problems, and these methods may be suitable across varying types of classification problems and combined to create an overall voting system for detecting classification accuracy [25–27]. This allows researchers to produce ensembles based on several algorithm decisions for blocked pairs. In effect, training several algorithms allows further triangulation of identifying matched pairs between data sources.

### Limitations

There are several limitations to note in our current work. First, the variation in unique name values available in our simulation was lower than the variation encountered in real-world data (i.e., birth cohort data showed more unique names when relative size was considered). This seems to be a product of the data generation functions used, and future work should test how increasing this source of variance affects results. Second, our simulation linkage used person and address information, whereas our applied analysis included guardian information in addition to the fields used in the simulation analysis. The introduction of more fields for comparing record pairs allows for a more nuanced and accurate scoring model based on increasing the number of points of comparison and thus, points where records with more than one match pair can differ.

### Conclusion

The PRLF record linkage solution encapsulates all aspects of data cleaning, blocking, match probability modeling and scoring, and final linkage production. Our linkage solution, PRLF, is adaptable and allows researchers to adjust each step to best fit their needs. That is, if accuracy is of highest import (e.g., researchers would only like to identify true positives and reduce false positives), then a high threshold for selecting a match should be prioritized. These requirements will vary by use case, and PRLF has many ways to handle a wide range of applications for research making use of administrative data.

## Supporting information

S1 Fig Step by step workflow for PRLF model training and data scoring. The inner two columns show the two workflows of PRLF: 1. Assigning match probabilities to potential shared pairs between two datasets, and 2. Generating models based on user-provided training data which can be used as score functions for Workflow 1.
(TIF)

S1 Text Probabilistic record linkage for families (PRLF) workflow process. (DOCX)

S2 Text Simulated dataset generation. (DOCX)

S3 Text Sample of features generated during feature extraction. (DOCX)

S4 Text Simulated data linkage for 10,000 and 100,000. (DOCX)

S1 Table Sample of features generated when comparing records from two different sources. (DOCX)

S2 Table Fit statistics for scored XGBoost models with 10,000 rows per dataset. (DOCX)

S3 Table Fit statistics for scored XGBoost models with 100,000 rows per dataset. (DOCX)

S4 Table Overlap of LinkPlus and ChoiceMaker matched pairs with varied thresholds for ChoiceMaker. (DOCX)
