# Supplementary figures and images for: An open-source probabilistic record linkage process for records with family-level information: Simulation study and applied analysis
Source: PLoS One. 2023 Oct 20;18(10):e0291581. doi: 10.1371/journal.pone.0291581 (PMC10588881; doi:10.1371/journal.pone.0291581)

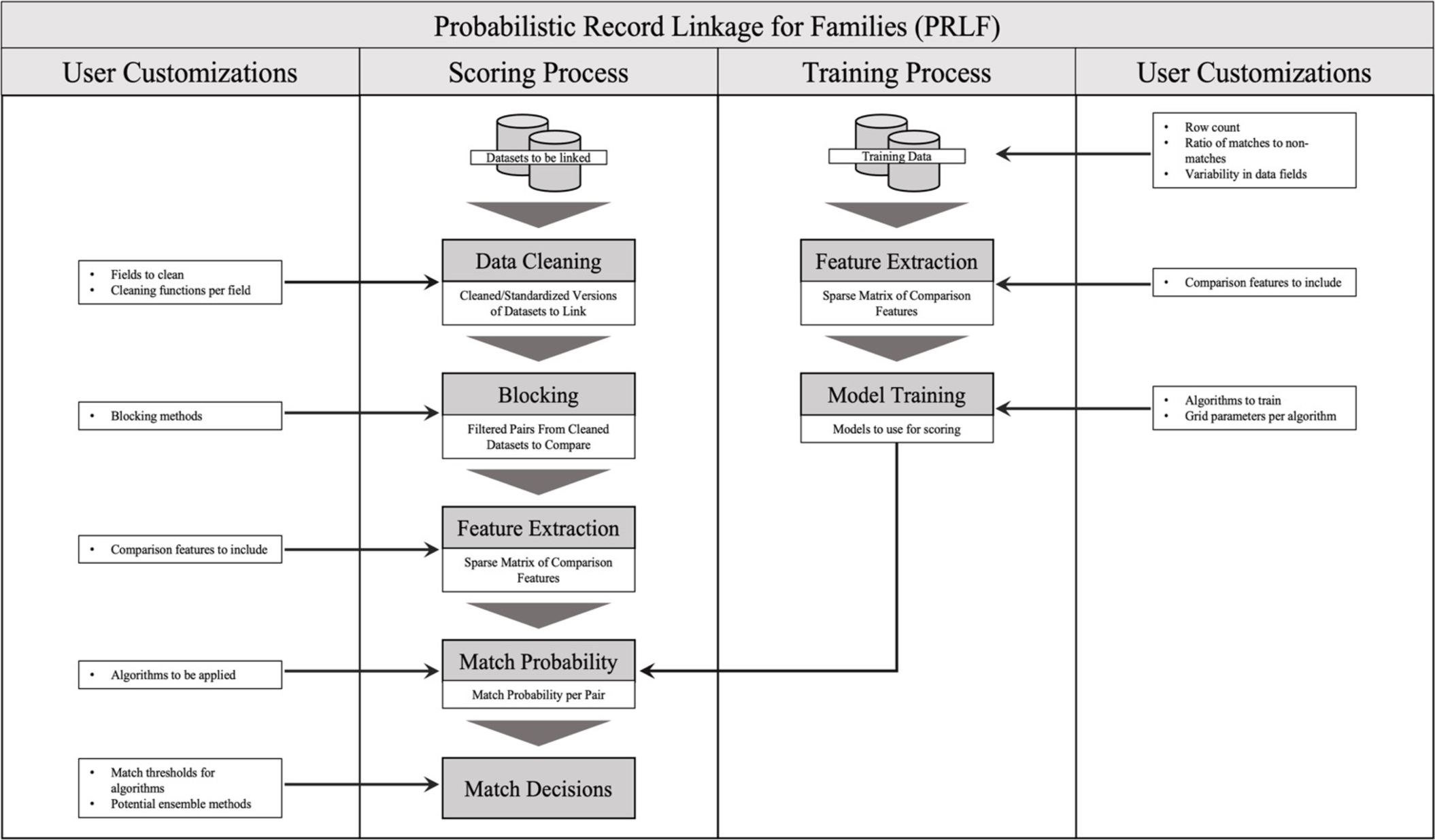

Supplement: S1 Fig — The inner two columns show the two workflows of PRLF: 1. Assigning match probabilities to potential shared pairs between two datasets, and 2. Generating models based on user-provided training data which can be used as score functions for Workflow 1. (TIF) [file pone.0291581.s001.tif]
