# Supplementary material for: An open-source probabilistic record linkage process for records with family-level information: Simulation study and applied analysis
Source: PLoS One. 2023 Oct 20;18(10):e0291581. doi: 10.1371/journal.pone.0291581 (PMC10588881; doi:10.1371/journal.pone.0291581)
Supplement: S1 Text — (DOCX) [file pone.0291581.s002.docx]

**Text S1. Probabilistic Record Linkage for Families (PRLF) Workflow Process**

The inner two columns show the two workflows of PRLF: 1. Assigning match probabilities to potential shared pairs between two datasets, and 2. Generating models based on user-provided training data which can be used as score functions for Workflow 1.

Unless models are already provided or generated, users must first go through the Training Process. The first step in this process is providing training data – the individual level PII of records which are known matches or non-matches. Users can customize the number of rows as well as the ratio of matches to non-matches (i.e., the number of match or non-match pairs), the available PII fields which can be compared, and the variability of PII (e.g., number of unique first names). It is recommended that the user provide training data which is as representative of the datasets they will be performing record linkage on as possible. Next, the training data undergoes Feature Extraction, which generates a matrix of 0/1 (False/True) comparison features per training data row, for all comparisons specified by the user. This matrix is then fed to the Model Training step for which users can specify the algorithms they wish to train, as well as the hyperparameters which the users want to optimize the model for.

Once trained models have been procured, the Scoring Process can be initiated. The first step in this process is cleaning both datasets, which standardizes column names, date formats, and applies other user specified cleaning functions to user specified columns (e.g., capitalizing all name columns, removing specific characters from all address fields, converting invalid zip codes to blank). The cleaned datasets are then blocked according to user defined algorithms to limit the number of rows that undergo Feature Extraction. After Feature Extraction, trained models are used to assign match probabilities to each potential pair. The final step in this process is the Match Decision, in which users can set a match probability threshold per model or models for what to consider a match and non-match.
