# Supplementary material for: An open-source probabilistic record linkage process for records with family-level information: Simulation study and applied analysis
Source: PLoS One. 2023 Oct 20;18(10):e0291581. doi: 10.1371/journal.pone.0291581 (PMC10588881; doi:10.1371/journal.pone.0291581)
Supplement: S2 Text — (DOCX) [file pone.0291581.s003.docx]

**Text S2. Simulated Dataset Generation**

*Explanation:*

Two datasets were generated per simulation run, with each pair of datasets having a unique set of parameters. The parameters included dataset size, percent overlap (e.g., percentage of records found both datasets), and percent error in each of the datasets. For example, if the parameters for a particular run were 100,000 for dataset size, 5% overlap, and 5% error rate, two datasets of 100,000 rows each would be created, with 5,000 records in each of the datasets having the same information, and 5,000 rows having some form of data degradation (e.g., first name is missing, or birth year and birth dates are swapped).

Datasets were creating using the Python package Faker, which generated the following information for records based on a random seed:

- First Name
- Middle Name
- Last Name
- Street Address
- Street Name
- City Name

In each of the two datasets, 50% of the records were created to have first and middle names commonly associated with females and 50% of the records were created to have first and middle names commonly associated with males (as determined by the Faker package). Consequently, the Gender field was initially set to be 50% Female and 50% Male.

To better reflect real world data, 5% of all records created did not have a middle name and 5% of all records created had a two-part first name (e.g., Mary Kate).

A Date of Birth field was created for every record which was a randomly selected date from the year 2016.

After generation of the above columns by the Faker package, additional columns were added to every row in each of the two datasets:

- SourceID – an alphanumeric unique identifier for each record
- State Name – set as “California” for all records
- Zip Code – a random number from the range 90001-96162 (the range of zip codes in California)

In order to mimic data errors seen in practice, each dataset in the linkage pair would have a percentage of rows selected for which fields in that row would have some form of data degradation introduced. Each row in the selection would first have a random subset of information blocks (e.g., Name, Birthdate, Address) selected for which degradation would be applied. Within each information block, one function from the following list would be applied:

Name:

- Keep only first character of first name
- Keep only first character of last name
- Drop first name
- Drop last name
- Drop middle name
- Swap first name and last name
- Delete last character of first name
- Delete last character of last name
- Delete last two characters of first name
- Delete last two characters of last name
- Concatenate first and last names in first name field
- Concatenate first and middle names in first name field
- Replace first name with a commonly used unknown names (e.g., UNK, CHILD, ADOPTED)
- Replace first name with random first name in pre-determined list
- Replace last name with random first name in pre-determined list

Gender:

- Swap Gender (e.g., F becomes M or M becomes F)
- Drop gender
- Replace gender with random element from list: Blank, Unknown, Invalid, T, U, NA

DOB:

- Replace day portion with a random number between 1 and 30
- Replace month portion with a random number between 1 and 12
- Replace year portion with random 4 digit number
- Swap the month and day portions
- Swap the last two characters of year
- Swap the two characters of day
- Swap the two digits of month
- Replace day portion with 00
- Replace year portion with 0000
- Replace entire dob with random element from list: Blank, Unknown, Invalid

Address:

- Drop street address
- Drop street number
- Drop street suffix
- Drop street name
- Reorder street number
- Drop city
- Keep only first character of city
- Drop last two characters of city
- Drop last character of city
- Drop zip code
- Replace zip code with 90000
- Replace zip code with 00000
- Replace zip code with random zip code between 90001 and 96161
- Re-order zip code
- Replace street suffix with random street suffix
- Replace street number with random number of same character length
- Keep first digit of street number and replace the rest with a random number

After error introduction, both datasets would undergo a cleaning process identical to that of the production PRLF environment, which consisted of the following functions:

- Capitalizing all strings
- Removing any special characters from fields
- Removing commonly used error or blank strings (e.g., Blank, Unknown, Withheld) from fields
- Removing erroneous zip code 99999
- Removing Suffixes from name fields (e.g., JR, II)

The dataset generation parameters across all the simulation runs are as follows:

- Dataset Sizes: 10,000; 50,000; 100,000
- Percent Overlap: 1%; 3%; 5%; 10%; 20%; 40%; 50%
- Error Rate: 0%; 2%; 5%; 10%; 20%; 30%; 40%

Thus, a total of 168 dataset pairs were created, with each pair having a unique combination of the above three parameters.

*Code:*

Python code used to create simulated datasets. First lines of code are identified to provide different simulation parameters over each iteration.

Main Workflow:

#### Import Packages

import pandas as pd

import numpy as np

import faker

from faker import Faker

import random

from random import choices

import math

from datetime import date

import data_jitter_functions as djf

#### User Inputs

# output path

out_path = "OUTPUTFILEPATH"

# Number of records each pair will have

df_size_list = [25000, 50000, 100000, 250000, 500000, 1000000]

# Percentage of overlap between both records (with reference to df1)

perc_overlap_list = [0, .01, .02, .03, .04, .05, .07, .1, .15, .20, .25, .30, .4, .5, .6, .7]

# percentage of rows to add jitter to

perc_rows_list = [0, .01, .02, .03, .04, .05, .07, .1, .15, .20, .25, .30, .4, .5]

# Percentage of records to have a multi part name

perc_multi_part = .05

# Percentage of records to not have a middle

perc_no_mname = .05

# Location of Unique First and Last Names, used in data degradation process

unq_fn_path = "FILEPATH"

unq_ln_path = "FILEPATH"

# Information fields to add data degradation

#info_cols = ['name', 'ssn', 'gender', 'dob', 'address']

info_cols = ['name', 'ssn', 'gender', 'dob', 'address']

#### Create Simulated Data

if __name__ == "__main__":

for df_size in df_size_list:

for perc_overlap in perc_overlap_list:

for perc_rows in perc_rows_list:

# filenames of dfs

df1_name = 'df1_' + str(df_size) + '_' + str(perc_overlap) + '_' + str(perc_rows)

df2_name = 'df2_' + str(df_size) + '_' + str(perc_overlap) + '_' + str(perc_rows)

# row count

df1_rows = df_size

df2_rows = df_size

# set seeds based on df_size

# set seed for female

fseed = df_size

# set seed for male

mseed = df_size + 1

# set seed for multipart name

mpname_seed = df_size + 2

# set seed for no mname

nomname_seed = df_size + 3

# set seed for new female middle

new_fmname = df_size + 4

# set seed for new male middle

new_mmname = df_size + 5

# set seed for re-ordering df

order_seed = df_size + 6

# set seed for jitter

jit_seed = df_size + 7

# initialize

fake = Faker()

# today's date

today = str(date.today()).replace("-","_")

tot_rows = int(df1_rows + df2_rows - perc_overlap*df1_rows)

nrow_f = int(tot_rows/2)

nrow_m = int(tot_rows/2)

#### Female Records

Faker.seed(fseed)

random.seed(fseed)

female_records = [

{'sourceid': '',

'name': fake.name_female(),

'middle': fake.first_name_female(),

'gender': '',

'dob': fake.date_between_dates(date_start = pd.to_datetime("2016-01-01"), date_end = pd.to_datetime("2016-12-31")),

'ssn': fake.ssn(),

'addressid': '',

'street': fake.street_address(),

'street_name': fake.street_name(),

'street_suffix': fake.street_suffix(),

'city': fake.city(),

'state': ''}

for x in range(nrow_f)]

df_f = pd.DataFrame(female_records)

df_f['sourceid'] = np.arange(len(df_f))

df_f['sourceid'] = df_f['sourceid'].astype(str)

df_f['sourceid'] = 'f_' + df_f['sourceid']

df_f['gender'] = 'F'

df_f['state'] = 'CA'

df_f['zip'] = np.random.randint(90001, 96162, df_f.shape[0])

df_f['addressid'] = df_f['sourceid'].astype(str) + '_a'

df_f.head()

#### Male Records

Faker.seed(mseed)

random.seed(mseed)

male_records = [

{'sourceid': '',

'name': fake.name_male(),

'middle': fake.first_name_male(),

'gender': '',

'dob': fake.date_between_dates(date_start = pd.to_datetime("2016-01-01"), date_end = pd.to_datetime("2016-12-31")),

'ssn': fake.ssn(),

'addressid': '',

'street': fake.street_address(),

'street_name': fake.street_name(),

'city': fake.city(),

'state': ''}

for x in range(nrow_m)]

df_m = pd.DataFrame(male_records)

df_m['sourceid'] = np.arange(len(df_m))

df_m['sourceid'] = df_m['sourceid'].astype(str)

df_m['sourceid'] = 'm_' + df_m['sourceid']

df_m['gender'] = 'M'

df_m['state'] = 'CA'

df_m['zip'] = np.random.randint(90001, 96162, df_m.shape[0])

df_m['addressid'] = df_m['sourceid'].astype(str) + '_a'

df_m.head()

#### Combine

df = df_f.append(df_m)

#### Cleaning/Standardization

# remove prefixes/suffixes, special characters, and separate name into two columns

df['name'] = df['name'].str.upper()

df['name'] = df['name'].str.replace('-', ' ', regex = False) # replace dash with space

df['name'] = df['name'].str.replace('[^A-Za-z +]', ' ', regex = True) # all special characters except space

df['name'] = df['name'].str.strip() # remove trailing and leading spaces

pattern = "DR |MRS |MR |MS | MD| DDS| PHD| DVM"

df['name'] = df['name'].str.replace(pattern, '', regex = True)

df['name'] = df['name'].str.replace(' ', '', regex = False)

df['name'] = df['name'].str.strip()

df[['cfirstname', 'clastname']] = df['name'].str.split(" ", 1, expand = True)

# capitalize middle name

df['middle'] = df['middle'].str.upper()

#### Combine % of first and middle names defined above

random.seed(mpname_seed)

sourceid_combinenames = random.sample(set(df.sourceid), math.ceil(perc_multi_part*df.shape[0]))

df_combinednames = df[df.sourceid.isin(sourceid_combinenames)]

df_combinednames['cfirstname'] = df_combinednames['cfirstname'] + ' ' + df_combinednames['middle']

new_fmiddle = df_combinednames[df_combinednames['gender'] == 'F'].shape[0]

Faker.seed(new_fmname)

female_records = [

{'middle': fake.first_name_female()}

for x in range(new_fmiddle)]

new_fmiddle = pd.DataFrame(female_records)

new_mmiddle = df_combinednames[df_combinednames['gender'] == 'M'].shape[0]

Faker.seed(new_mmname)

male_records = [

{'middle': fake.first_name_male()}

for x in range(new_mmiddle)]

new_mmiddle = pd.DataFrame(male_records)

df_combinednames.middle[df_combinednames['gender'] == 'F'] = new_fmiddle['middle']

df_combinednames.middle[df_combinednames['gender'] == 'M'] = new_mmiddle['middle']

# dataframe to keep as is

df_keep = df[-df.sourceid.isin(sourceid_combinenames)]

df = pd.concat([df_keep, df_combinednames])

df = df.sort_values(by = ['sourceid'])

#### Drop % of middle names defined above

random.seed(nomname_seed)

sourceid_drop_mname = random.sample(set(df.sourceid), math.ceil(perc_no_mname*df.shape[0]))

# dataframe to drop mname

df_drop_mname = df[df.sourceid.isin(sourceid_drop_mname)]

df_drop_mname['middle'] = ''

# dataframe to keep as is

df_keep = df[-df.sourceid.isin(sourceid_drop_mname)]

df = pd.concat([df_keep, df_drop_mname])

df = df.sort_values(by = ['sourceid'])

#### More Cleaning

df['middle'] = df['middle'].str.upper()

# street cleaning

df['street'] = df['street'].str.split().str[0]

df['street_name'] = df['street_name'].str.upper()

df['street_name'] = df['street_name'].str.replace(r"\W+|_", ' ', regex = True)

pattern = "UNK|UNKNOWN|WITHHELD|XXX"

df['street_name'].str.replace(pattern, '', regex = True)

df['street_name'] = df['street_name'].str.strip()

street_suffix = choices(['Street', 'St.', 'Boulevard', 'Blvd.', 'Lane', 'Ln.', 'Avenue', 'Ave.', 'Road', 'Rd.', 'Corner', 'Court', 'Terrace', ''], k = df.shape[0])

df['street_suffix'] = street_suffix

df['street_suffix'] = df['street_suffix'].str.upper()

df['street'] = df['street'] + ' ' + df['street_name'] + ' ' + df['street_suffix']

# city cleaning

df['city'] = df['city'].str.upper()

df['city'] = df['city'].str.replace(r"\W+|_", ' ', regex = True)

pattern = "UNK|UNKNOWN|WITHHELD|1|2|3|4|5|6|7|8|9|0|XXX"

df['city'].str.replace(pattern, '', regex = True)

df['city'] = df['city'].str.strip()

df = df[['sourceid', 'cfirstname', 'middle', 'clastname', 'dob', 'ssn', 'gender', 'addressid', 'street', 'city', 'state', 'zip']]

df.columns = ['sourceid', 'cfirstname', 'cmiddlename', 'clastname', 'cbirthdate', 'cssn', 'cgender', 'addressid', 'cstreet1', 'ccity', 'cstate', 'czip']

#### Splitting Data

random.seed(order_seed)

df = df.sample(frac = 1)

df1 = df.iloc[0:df1_rows,:].copy()

df2 = df.iloc[df1_rows-int(perc_overlap*df1_rows):,:].copy()

df2['sourceid'] = 'df2_' + str(df_size) + '_' + str(perc_overlap) + '_' + str(perc_rows) + '_' + df2['sourceid']

df1['sourceid'] = 'df1_' + str(df_size) + '_' + str(perc_overlap) + '_' + str(perc_rows) + '_' + df1['sourceid']

df1['addressid'] = df1['sourceid'].astype(str) + '_a'

df2['addressid'] = df2['sourceid'].astype(str) + '_a'

### Jitter Data

def jit(df):

func_map = {

'name': [djf.abbr_fn, djf.abbr_ln, djf.drop_fn, djf.drop_mn, djf.drop_ln, djf.swap_name, djf.dlc_fname, djf.dlc_lname, djf.dl2c_fname, djf.dl2c_lname, djf.combine_names, djf.unk_fname, djf.rand_fname, djf.rand_lname, djf.comb_firstmiddle],

'ssn': [djf.drop_ssnfirst, djf.dlc_ssn, djf.dl2c_ssn, djf.drop_ssn, djf.string_ssn, djf.string_ssn, djf.invalid_ssn],

'gender': [djf.drop_gender, djf.swap_gender, djf.rand_gender],

'dob': [djf.random_day, djf.random_month, djf.random_year, djf.swap_daymonth, djf.swap_l2cyear, djf.swap_dayc, djf.swap_monthc, djf.zero_day, djf.zero_month, djf.zero_year, djf.string_dob],

'address': [djf.drop_addr, djf.drop_streetno, djf.drop_streetsuf, djf.drop_streetname, djf.shuffle_streetno, djf.drop_city, djf.init_city, djf.drop2_city, djf.drop1_city, djf.drop_zip, djf.nine_zip, djf.zero_zip, djf.rand_zip, djf.shuf_zip, djf.rand_streetsuf, djf.rand_streetno, djf.rand_streetnokeepfirst]}

# randomly select rows from dataframe to add jitter to

jitter_id = random.sample(set(df.sourceid), math.ceil(perc_rows*df.shape[0]))

# dataframe to apply jitter to

df_jit = df[df.sourceid.isin(jitter_id)]

# dataframe to keep as is

df_keep = df[-df.sourceid.isin(jitter_id)]

def jit_log(df, perc_rows, info_cols, func_map):

random.seed(df['sourceid'])

seed = df['sourceid']

num_info = random.choice(range(1,len(info_cols)+1))

jit_info = random.sample(info_cols, num_info)

func_dict = {}

for info in jit_info:

func_dict[info] = random.choice(func_map[info])

df['seed'] = seed

df['jit_info'] = jit_info

df['func_appl'] = func_dict

return df

df_jit = df_jit.apply(lambda row: jit_log(row, perc_rows, info_cols, func_map), axis = 1)

df_jit[list(df)] = df_jit[list(df)].astype(str)

new_df = pd.DataFrame()

for i, row in df_jit.iterrows():

new_row = row

for jit_func in row['func_appl'].values():

new_row = pd.DataFrame(jit_func(row)).transpose()

new_df = pd.concat([new_df, new_row])

return(pd.concat([df_keep, new_df]))

df1 = jit(df1)

df2 = jit(df2)

df1[list(df1)]= df1[list(df1)].astype(str)

df2[list(df2)]= df2[list(df2)].astype(str)

df1.to_csv(path_out, index = False)

df2.to_csv(path_out, index = False)

Helper Functions:

#### Import Packages

import pandas as pd

import numpy as np

import datetime as dt

import re

import random

from random import randint

import jellyfish

import random

from data_generation_jitter_cleaning import unq_fn_path, unq_ln_path

#### Define Functions

unq_fn = pd.read_csv(unq_fn_path)

unq_ln = pd.read_csv(unq_ln_path)

street_suf = ['Street', 'St.', 'Boulevard', 'Blvd.', 'Lane', 'Ln.', 'Avenue', 'Ave.', 'Road', 'Rd.', 'Corner', 'Court', 'Terrace', '']

def random_n_digits(n):

range_start = 10**(n-1)

range_end = (10**n)-1

return randint(range_start, range_end)

#### Name

def abbr_fn(df):

df['cfirstname'] = df['cfirstname'][:1]

return df

def abbr_ln(df):

df['clastname'] = df['clastname'][:1]

return df

def drop_fn(df):

df['cfirstname'] = ''

return df

def drop_ln(df):

df['clastname'] = ''

return df

def drop_mn(df):

df['cmiddlename'] = ''

return df

def swap_name(df):

cfirstname = df['cfirstname']

clastname = df['clastname']

df['cfirstname'] = clastname

df['clastname'] = cfirstname

return df

def dlc_fname(df):

df['cfirstname'] = df['cfirstname'][:-1]

return df

def dlc_lname(df):

df['clastname'] = df['clastname'][:-1]

return df

def dl2c_fname(df):

df['cfirstname'] = df['cfirstname'][:-2]

return df

def dl2c_lname(df):

df['clastname'] = df['clastname'][:-2]

return df

def combine_names(df):

df['cfirstname'] = df['cfirstname'] + df['clastname']

df['clastname'] = ''

return df

def unk_fname(df):

random.seed(df['seed'])

df['cfirstname'] = random.choice(["BABYGIRL", "BABY GIRL", "BABYBOY", "BABY BOY", "ADOPT", "ADOPTED", "UNK", "UNKNOWN", "BABY", "BOY", "GIRL", "CHILD", "FEMALE", "TWIN", "UNBORN", "DECEASED", "NAME", "DAUGHTER"])

return df

def rand_fname(df):

random.seed(df['seed'])

df['cfirstname'] = random.choice(list(unq_fn.cfirstname))

return df

def rand_lname(df):

random.seed(df['seed'])

df['clastname'] = random.choice(list(unq_ln.clastname))

return df

def comb_firstmiddle(df):

df['cfirstname'] = df['cfirstname'] + ' ' + df['cmiddlename']

return df

#### SSN

def drop_ssnfirst(df):

df['cssn'] = str(df['cssn'][1:])

return df

def dlc_ssn(df):

df['cssn'] = str(df['cssn'][:-1])

return df

def dl2c_ssn(df):

df['cssn'] = str(df['cssn'][:-2])

return df

def drop_ssn(df):

df['cssn'] = ''

return df

def string_ssn(df):

random.seed(df['seed'])

df['cssn'] = random.choice(['BLANK', 'UNKNOWN', 'INVALID'])

return df

def invalid_ssn(df):

random.seed(df['seed'])

df['cssn'] = random.choice(['123456789', '000000000', '111111111', '999999999'])

return df

def random_ssn(df):

random.seed(df['seed'])

df['cssn'] = random.choice(range(100000000, 999999999))

return df

#### Gender

def swap_gender(df):

if df['cgender'] == 'M':

df['cgender'] = 'F'

if df['cgender'] == 'F':

df['cgender'] = 'M'

return df

def drop_gender(df):

df['cgender'] = ''

return df

def rand_gender(df):

random.seed(df['seed'])

df['cgender'] = random.choice(['BLANK', 'UNKNOWN', 'INVALID', 'T', 'U', 'NA'])

return df

#### DOB

def random_day(df):

random.seed(df['seed'])

df['cbirthdate'] = df['cbirthdate'][:-2] + str(random.choice(range(1,30))).zfill(2)

return df

def random_month(df):

random.seed(df['seed'])

df['cbirthdate'] = df['cbirthdate'][:4] + str(random.choice(range(1,13))).zfill(2) + df['cbirthdate'][-2:]

return df

def random_year(df):

random.seed(df['seed'])

df['cbirthdate'] = df['cbirthdate'][:2] + str(random.choice(range(1,100))).zfill(2) + df['cbirthdate'][4:]

return df

def swap_daymonth(df):

df['cbirthdate'] = df['cbirthdate'][:5] + df['cbirthdate'][8:10] + df['cbirthdate'][4:7]

return df

def swap_l2cyear(df):

df['cbirthdate'] = df['cbirthdate'][:2] + df['cbirthdate'][3] + df['cbirthdate'][2] + df['cbirthdate'][4:]

return df

def swap_dayc(df):

df['cbirthdate'] = df['cbirthdate'][:-2] + df['cbirthdate'][-1] + df['cbirthdate'][-2]

return df

def swap_monthc(df):

df['cbirthdate'] = df['cbirthdate'][:5] + df['cbirthdate'][6] + df['cbirthdate'][5] + df['cbirthdate'][-3:]

return df

def zero_day(df):

df['cbirthdate'] = df['cbirthdate'][:-2] + '00'

return df

def zero_month(df):

random.seed(df['seed'])

df['cbirthdate'] = df['cbirthdate'][:4] + '00' + df['cbirthdate'][-2:]

return df

def zero_year(df):

random.seed(df['seed'])

df['cbirthdate'] = '0000' + df['cbirthdate'][4:]

return df

def string_dob(df):

random.seed(df['seed'])

df['cbirthdate'] = random.choice(['BLANK', 'UNKNOWN', 'INVALID'])

return df

#### Address

def drop_addr(df):

df['cstreet1'] = ''

return df

def drop_streetno(df):

df['cstreet1'] = ' '.join([str(item) for item in df['cstreet1'].split()[1:]])

return df

def drop_streetsuf(df):

df['cstreet1'] = ' '.join([str(item) for item in df['cstreet1'].split()[0:-1]])

return df

def drop_streetname(df):

df['cstreet1'] = df['cstreet1'].split()[0] + ' ' + df['cstreet1'].split()[-1]

return df

def shuffle_streetno(df):

random.seed(df['seed'])

df['cstreet1'] = ''.join(random.sample(df['cstreet1'].split()[0], len(df['cstreet1'].split()[0]))) + ' ' + ' '.join([str(item) for item in df['cstreet1'].split()[1:]])

return df

def drop_city(df):

df['ccity'] = ''

return df

def init_city(df):

df['ccity'] = df['ccity'][0]

return df

def drop2_city(df):

df['ccity'] = df['ccity'][:len(df['ccity'])-2]

return df

def drop1_city(df):

df['ccity'] = df['ccity'][:len(df['ccity'])-1]

return df

def drop_zip(df):

df['czip'] = ''

return df

def nine_zip(df):

df['czip'] = '90000'

return df

def zero_zip(df):

df['czip'] = '00000'

return df

def rand_zip(df):

random.seed(df['seed'])

df['czip'] = random.choice(range(90001, 96162))

return df

def shuf_zip(df):

random.seed(df['seed'])

temp = [*str(df['czip'])][1:]

random.shuffle(temp)

df['czip'] = (''.join([str(elem) for elem in [*[*str(df['czip'])][0],*temp]]))

return df

def rand_streetsuf(df):

random.seed(df['seed'])

df['cstreet1'] = ' '.join([str(item) for item in df['cstreet1'].split()[0:-1]]) + ' ' + random.choice(list(set(street_suf) - set(df['cstreet1'].split()[-1])))

return df

def rand_streetno(df):

random.seed(df['seed'])

df['cstreet1'] = str(random_n_digits(len(df['cstreet1'].split()[0]))) + ' ' + ' '.join([str(item) for item in df['cstreet1'].split()[1:]])

return df

def rand_streetnokeepfirst(df):

random.seed(df['seed'])

df['cstreet1'] = str(df['cstreet1'].split()[0][0]) + str(random_n_digits(len(df['cstreet1'].split()[0])-1)) + ' ' + ' '.join([str(item) for item in df['cstreet1'].split()[1:]])

return df
