# Supplementary material for: An open-source probabilistic record linkage process for records with family-level information: Simulation study and applied analysis
Source: PLoS One. 2023 Oct 20;18(10):e0291581. doi: 10.1371/journal.pone.0291581 (PMC10588881; doi:10.1371/journal.pone.0291581)
Supplement: S3 Text — (DOCX) [file pone.0291581.s004.docx]

**Text S3. Sample of Features Generated During Feature Extraction**

In order for a feature to be generated, the respective field must exist in both datasets (i.e., a Zip Code Exact Match feature will only be generated if Zip Code exists for both records being compared). All features are coded as binary (0/1), and the converse feature is automatically generated where appropriate (i.e., a Zip Code Not Exact Match feature will be generated and have the opposite value of Zip Code Exact Match).
