# Supplementary material for: An open-source probabilistic record linkage process for records with family-level information: Simulation study and applied analysis
Source: PLoS One. 2023 Oct 20;18(10):e0291581. doi: 10.1371/journal.pone.0291581 (PMC10588881; doi:10.1371/journal.pone.0291581)
Supplement: S4 Text — (DOCX) [file pone.0291581.s005.docx]

**Text S4. Simulated Data Linkage for 10,000 and 100,000**

Two additional sets of simulated data were created to test if any differences in performance could be detected as the scale of data was changed (smaller versus larger). Table B presents the results of simulated datasets with 10,000 rows simulated per pair. Table C presents results with simulated datasets with 100,000 rows per pair of datasets. The trends noted in Table 3 hold for these two additional comparisons. There is a notable increase in accuracy as the dataset size decreases. This could be due to the lower variability in the number of unique names present in simulated data compared to administrative data. As the simulation row count increases the list of possible names is relatively constant and overall variability decreases. From this, match pairs are more difficult to separate as the amount of unique information per a row is relatively lower compared what we would see in an applied setting.
