# Supplementary material for: An open-source probabilistic record linkage process for records with family-level information: Simulation study and applied analysis
Source: PLoS One. 2023 Oct 20;18(10):e0291581. doi: 10.1371/journal.pone.0291581 (PMC10588881; doi:10.1371/journal.pone.0291581)
Supplement: S1 Table — (DOCX) [file pone.0291581.s006.docx]

| Table S1. Sample of features generated when comparing records from two different sources. | | |
| --- | --- | --- |
| Child Specific Features* | Address Specific Features* | Guardian Specific Features |
| • First Name Exact Match • Last Name Soundex Match • Last Name Levenshtein Distance Less Than 5% of name length • Birth Month and Birth Date switched • Birth Year Exact Match | • City Exact Match • Least Common String of Street Address at least 24 characters • City Name Soundex Match • Zip Code Exact Match • Street Address Edit Distance less than 5% of street address length | • Mother First Name Exact Match • Father Last Name Soundex Match • Father Last Name Levenshtein Distance Less Than 5% of name length • Mother Last Name and Father Last Name Exact Match • Birth Year Exact Match |
