# Supplementary material for: An open-source probabilistic record linkage process for records with family-level information: Simulation study and applied analysis
Source: PLoS One. 2023 Oct 20;18(10):e0291581. doi: 10.1371/journal.pone.0291581 (PMC10588881; doi:10.1371/journal.pone.0291581)
Supplement: S2 Table — (DOCX) [file pone.0291581.s007.docx]

| Table S2. Fit Statistics For Scored XGBoost Models with 10,000 Rows Per Dataset | | | | | | | |  |
| --- | --- | --- | --- | --- | --- | --- | --- | --- |
| A. Accuracy (True Positives and True Negatives) | | | | |  |  |  |  |
| Overlap | Proportion of Rows with Errors | | | | | | | |
|  | 0 | 0.03 | 0.05 | 0.1 | 0.15 | 0.2 | 0.3 | 0.4 |
| 0.01 | 0.904 | 0.913 | 0.910 | 0.916 | 0.916 | 0.914 | 0.936 | 0.941 |
| 0.03 | 0.918 | 0.908 | 0.918 | 0.924 | 0.921 | 0.923 | 0.935 | 0.940 |
| 0.05 | 0.911 | 0.920 | 0.916 | 0.924 | 0.926 | 0.932 | 0.937 | 0.944 |
| 0.1 | 0.927 | 0.923 | 0.923 | 0.930 | 0.931 | 0.935 | 0.936 | 0.946 |
| 0.2 | 0.940 | 0.948 | 0.941 | 0.943 | 0.943 | 0.940 | 0.944 | 0.949 |
| 0.4 | 0.965 | 0.968 | 0.964 | 0.959 | 0.957 | 0.960 | 0.951 | 0.946 |
| 0.5 | 0.979 | 0.976 | 0.974 | 0.973 | 0.971 | 0.965 | 0.954 | 0.944 |
| B. Sensitivity | |  |  |  |  |  |  |  |
| Overlap | Proportion of Rows with Errors | | | | | | | |
|  | 0 | 0.03 | 0.05 | 0.1 | 0.15 | 0.2 | 0.3 | 0.4 |
| 0.01 | 1.000 | 1.000 | 1.000 | 1.000 | 0.988 | 0.988 | 0.970 | 0.955 |
| 0.03 | 1.000 | 0.997 | 1.000 | 0.996 | 0.984 | 0.969 | 0.966 | 0.938 |
| 0.05 | 1.000 | 1.000 | 0.991 | 0.989 | 0.985 | 0.987 | 0.984 | 0.936 |
| 0.1 | 1.000 | 0.998 | 0.996 | 0.986 | 0.985 | 0.980 | 0.965 | 0.940 |
| 0.2 | 1.000 | 0.999 | 0.992 | 0.991 | 0.983 | 0.976 | 0.964 | 0.950 |
| 0.4 | 1.000 | 0.998 | 0.996 | 0.989 | 0.981 | 0.980 | 0.961 | 0.941 |
| 0.5 | 1.000 | 0.999 | 0.995 | 0.989 | 0.986 | 0.979 | 0.960 | 0.943 |
| C. Specificity | |  |  |  |  |  |  |  |
| Overlap | Proportion of Rows with Errors | | | | | | | |
|  | 0 | 0.03 | 0.05 | 0.1 | 0.15 | 0.2 | 0.3 | 0.4 |
| 0.01 | 0.903 | 0.911 | 0.908 | 0.914 | 0.915 | 0.912 | 0.936 | 0.940 |
| 0.03 | 0.913 | 0.903 | 0.913 | 0.920 | 0.918 | 0.921 | 0.934 | 0.940 |
| 0.05 | 0.902 | 0.912 | 0.909 | 0.918 | 0.921 | 0.927 | 0.933 | 0.944 |
| 0.1 | 0.910 | 0.906 | 0.908 | 0.919 | 0.921 | 0.926 | 0.931 | 0.947 |
| 0.2 | 0.905 | 0.920 | 0.913 | 0.918 | 0.925 | 0.924 | 0.937 | 0.948 |
| 0.4 | 0.899 | 0.911 | 0.909 | 0.912 | 0.922 | 0.934 | 0.942 | 0.951 |
| 0.5 | 0.907 | 0.907 | 0.910 | 0.929 | 0.935 | 0.938 | 0.945 | 0.946 |
| D. Precision | |  |  |  |  |  |  |  |
| Overlap | Proportion of Rows with Errors | | | | | | | |
|  | 0 | 0.03 | 0.05 | 0.1 | 0.15 | 0.2 | 0.3 | 0.4 |
| 0.01 | 0.164 | 0.171 | 0.170 | 0.169 | 0.162 | 0.162 | 0.179 | 0.188 |
| 0.03 | 0.405 | 0.371 | 0.393 | 0.401 | 0.382 | 0.372 | 0.400 | 0.382 |
| 0.05 | 0.515 | 0.538 | 0.514 | 0.536 | 0.527 | 0.538 | 0.543 | 0.534 |
| 0.1 | 0.719 | 0.706 | 0.700 | 0.712 | 0.710 | 0.713 | 0.695 | 0.719 |
| 0.2 | 0.857 | 0.874 | 0.859 | 0.859 | 0.859 | 0.849 | 0.853 | 0.857 |
| 0.4 | 0.950 | 0.955 | 0.950 | 0.947 | 0.946 | 0.951 | 0.945 | 0.942 |
| 0.5 | 0.973 | 0.971 | 0.971 | 0.974 | 0.972 | 0.969 | 0.965 | 0.956 |
| E. F1 Score | |  |  |  |  |  |  |  |
| Overlap | Proportion of Rows with Errors | | | | | | | |
|  | 0 | 0.03 | 0.05 | 0.1 | 0.15 | 0.2 | 0.3 | 0.4 |
| 0.01 | 0.282 | 0.292 | 0.291 | 0.289 | 0.278 | 0.278 | 0.302 | 0.313 |
| 0.03 | 0.577 | 0.540 | 0.564 | 0.572 | 0.550 | 0.537 | 0.565 | 0.542 |
| 0.05 | 0.680 | 0.700 | 0.677 | 0.695 | 0.687 | 0.696 | 0.700 | 0.680 |
| 0.1 | 0.837 | 0.827 | 0.822 | 0.827 | 0.825 | 0.825 | 0.808 | 0.815 |
| 0.2 | 0.923 | 0.932 | 0.921 | 0.920 | 0.917 | 0.908 | 0.905 | 0.901 |
| 0.4 | 0.974 | 0.976 | 0.973 | 0.968 | 0.964 | 0.965 | 0.953 | 0.941 |
| 0.5 | 0.986 | 0.984 | 0.983 | 0.981 | 0.979 | 0.974 | 0.962 | 0.949 |
| F. Matches in Block | |  |  |  |  |  |  |  |
| Overlap | Proportion of Rows with Errors | | | | | | | |
|  | 0 | 0.03 | 0.05 | 0.1 | 0.15 | 0.2 | 0.3 | 0.4 |
| 0.01 | 1.000 | 0.960 | 0.960 | 0.870 | 0.810 | 0.820 | 0.670 | 0.660 |
| 0.03 | 1.000 | 0.960 | 0.920 | 0.883 | 0.830 | 0.753 | 0.687 | 0.590 |
| 0.05 | 1.000 | 0.984 | 0.940 | 0.902 | 0.824 | 0.792 | 0.730 | 0.594 |
| 0.1 | 1.000 | 0.983 | 0.938 | 0.879 | 0.844 | 0.782 | 0.686 | 0.597 |
| 0.2 | 1.000 | 0.982 | 0.946 | 0.882 | 0.829 | 0.781 | 0.695 | 0.603 |
| 0.4 | 0.999 | 0.980 | 0.940 | 0.888 | 0.837 | 0.799 | 0.689 | 0.592 |
| 0.5 | 1.000 | 0.975 | 0.946 | 0.889 | 0.839 | 0.779 | 0.683 | 0.599 |
| G. True Matches in Block | | |  |  |  |  |  |  |
| Overlap | Proportion of Rows with Errors | | | | | | | |
|  | 0 | 0.03 | 0.05 | 0.1 | 0.15 | 0.2 | 0.3 | 0.4 |
| 0.01 | 0.002 | 0.001 | 0.002 | 0.001 | 0.002 | 0.002 | 0.001 | 0.002 |
| 0.03 | 0.005 | 0.004 | 0.004 | 0.005 | 0.004 | 0.004 | 0.004 | 0.004 |
| 0.05 | 0.008 | 0.008 | 0.007 | 0.008 | 0.007 | 0.007 | 0.007 | 0.006 |
| 0.1 | 0.015 | 0.015 | 0.015 | 0.015 | 0.015 | 0.015 | 0.014 | 0.013 |
| 0.2 | 0.030 | 0.030 | 0.030 | 0.029 | 0.029 | 0.030 | 0.029 | 0.026 |
| 0.4 | 0.058 | 0.058 | 0.057 | 0.057 | 0.057 | 0.057 | 0.053 | 0.048 |
| 0.5 | 0.070 | 0.071 | 0.072 | 0.070 | 0.070 | 0.068 | 0.066 | 0.060 |
