# Supplementary material for: An open-source probabilistic record linkage process for records with family-level information: Simulation study and applied analysis
Source: PLoS One. 2023 Oct 20;18(10):e0291581. doi: 10.1371/journal.pone.0291581 (PMC10588881; doi:10.1371/journal.pone.0291581)
Supplement: S3 Table — (DOCX) [file pone.0291581.s008.docx]

| Table S3. Fit Statistics For Scored XGBoost Models with 100,000 Rows Per Dataset | | | | | | | |  |
| --- | --- | --- | --- | --- | --- | --- | --- | --- |
| A. Accuracy (True Positives and True Negatives) | | | | |  |  |  |  |
| Overlap | Proportion of Rows with Errors | | | | | | | |
|  | 0 | 0.03 | 0.05 | 0.1 | 0.15 | 0.2 | 0.3 | 0.4 |
| 0.01 | 0.612 | 0.617 | 0.628 | 0.640 | 0.656 | 0.671 | 0.703 | 0.735 |
| 0.03 | 0.629 | 0.629 | 0.642 | 0.657 | 0.667 | 0.681 | 0.711 | 0.741 |
| 0.05 | 0.641 | 0.647 | 0.656 | 0.665 | 0.681 | 0.693 | 0.721 | 0.746 |
| 0.1 | 0.675 | 0.681 | 0.689 | 0.699 | 0.708 | 0.720 | 0.739 | 0.763 |
| 0.2 | 0.748 | 0.749 | 0.753 | 0.759 | 0.763 | 0.766 | 0.779 | 0.791 |
| 0.4 | 0.866 | 0.866 | 0.861 | 0.859 | 0.857 | 0.853 | 0.849 | 0.852 |
| 0.5 | 0.911 | 0.909 | 0.906 | 0.899 | 0.894 | 0.887 | 0.882 | 0.873 |
| B. Sensitivity | |  |  |  |  |  |  |  |
| Overlap | Proportion of Rows with Errors | | | | | | | |
|  | 0 | 0.03 | 0.05 | 0.1 | 0.15 | 0.2 | 0.3 | 0.4 |
| 0.01 | 1.000 | 1.000 | 0.995 | 0.993 | 0.990 | 0.991 | 0.991 | 0.969 |
| 0.03 | 1.000 | 0.999 | 0.998 | 0.995 | 0.993 | 0.987 | 0.977 | 0.969 |
| 0.05 | 1.000 | 0.999 | 0.996 | 0.995 | 0.991 | 0.988 | 0.980 | 0.965 |
| 0.1 | 1.000 | 0.999 | 0.997 | 0.995 | 0.991 | 0.988 | 0.975 | 0.965 |
| 0.2 | 1.000 | 0.999 | 0.998 | 0.994 | 0.991 | 0.988 | 0.978 | 0.966 |
| 0.4 | 1.000 | 0.999 | 0.998 | 0.994 | 0.991 | 0.987 | 0.979 | 0.966 |
| 0.5 | 1.000 | 0.999 | 0.997 | 0.994 | 0.991 | 0.987 | 0.977 | 0.962 |
| C. Specificity | |  |  |  |  |  |  |  |
| Overlap | Proportion of Rows with Errors | | | | | | | |
|  | 0 | 0.03 | 0.05 | 0.1 | 0.15 | 0.2 | 0.3 | 0.4 |
| 0.01 | 0.605 | 0.610 | 0.622 | 0.634 | 0.651 | 0.666 | 0.699 | 0.732 |
| 0.03 | 0.608 | 0.609 | 0.623 | 0.639 | 0.651 | 0.667 | 0.700 | 0.733 |
| 0.05 | 0.606 | 0.613 | 0.624 | 0.636 | 0.655 | 0.670 | 0.702 | 0.733 |
| 0.1 | 0.604 | 0.613 | 0.625 | 0.641 | 0.657 | 0.674 | 0.704 | 0.738 |
| 0.2 | 0.610 | 0.615 | 0.627 | 0.647 | 0.665 | 0.676 | 0.711 | 0.740 |
| 0.4 | 0.614 | 0.624 | 0.633 | 0.659 | 0.680 | 0.696 | 0.728 | 0.767 |
| 0.5 | 0.616 | 0.624 | 0.644 | 0.667 | 0.690 | 0.708 | 0.749 | 0.775 |
| D. Precision | |  |  |  |  |  |  |  |
| Overlap | Proportion of Rows with Errors | | | | | | | |
|  | 0 | 0.03 | 0.05 | 0.1 | 0.15 | 0.2 | 0.3 | 0.4 |
| 0.01 | 0.044 | 0.044 | 0.043 | 0.043 | 0.043 | 0.042 | 0.043 | 0.040 |
| 0.03 | 0.127 | 0.125 | 0.126 | 0.126 | 0.122 | 0.121 | 0.117 | 0.113 |
| 0.05 | 0.201 | 0.201 | 0.199 | 0.196 | 0.194 | 0.191 | 0.188 | 0.183 |
| 0.1 | 0.356 | 0.355 | 0.355 | 0.352 | 0.342 | 0.340 | 0.327 | 0.319 |
| 0.2 | 0.584 | 0.580 | 0.578 | 0.572 | 0.561 | 0.552 | 0.535 | 0.518 |
| 0.4 | 0.830 | 0.828 | 0.819 | 0.812 | 0.804 | 0.792 | 0.771 | 0.756 |
| 0.5 | 0.896 | 0.894 | 0.889 | 0.879 | 0.871 | 0.859 | 0.844 | 0.823 |
| E. F1 Score | |  |  |  |  |  |  |  |
| Overlap | Proportion of Rows with Errors | | | | | | | |
|  | 0 | 0.03 | 0.05 | 0.1 | 0.15 | 0.2 | 0.3 | 0.4 |
| 0.01 | 0.084 | 0.085 | 0.083 | 0.083 | 0.082 | 0.081 | 0.082 | 0.077 |
| 0.03 | 0.225 | 0.222 | 0.223 | 0.223 | 0.218 | 0.215 | 0.209 | 0.202 |
| 0.05 | 0.334 | 0.335 | 0.331 | 0.328 | 0.324 | 0.321 | 0.315 | 0.308 |
| 0.1 | 0.525 | 0.524 | 0.524 | 0.520 | 0.508 | 0.506 | 0.490 | 0.480 |
| 0.2 | 0.737 | 0.734 | 0.732 | 0.726 | 0.716 | 0.708 | 0.692 | 0.675 |
| 0.4 | 0.907 | 0.906 | 0.900 | 0.894 | 0.888 | 0.879 | 0.863 | 0.848 |
| 0.5 | 0.945 | 0.943 | 0.940 | 0.933 | 0.927 | 0.919 | 0.906 | 0.887 |
| F. Matches in Block | |  |  |  |  |  |  |  |
| Overlap | Proportion of Rows with Errors | | | | | | | |
|  | 0 | 0.03 | 0.05 | 0.1 | 0.15 | 0.2 | 0.3 | 0.4 |
| 0.01 | 0.994 | 0.973 | 0.923 | 0.873 | 0.813 | 0.759 | 0.675 | 0.555 |
| 0.03 | 0.994 | 0.968 | 0.932 | 0.881 | 0.816 | 0.766 | 0.658 | 0.551 |
| 0.05 | 0.993 | 0.972 | 0.930 | 0.879 | 0.812 | 0.760 | 0.662 | 0.573 |
| 0.1 | 0.994 | 0.967 | 0.935 | 0.878 | 0.808 | 0.762 | 0.656 | 0.557 |
| 0.2 | 0.994 | 0.971 | 0.935 | 0.876 | 0.813 | 0.763 | 0.653 | 0.562 |
| 0.4 | 0.995 | 0.971 | 0.935 | 0.875 | 0.822 | 0.763 | 0.661 | 0.563 |
| 0.5 | 0.995 | 0.970 | 0.934 | 0.877 | 0.819 | 0.765 | 0.657 | 0.566 |
| G. True Matches in Block | | |  |  |  |  |  |  |
| Overlap | Proportion of Rows with Errors | | | | | | | |
|  | 0 | 0.03 | 0.05 | 0.1 | 0.15 | 0.2 | 0.3 | 0.4 |
| 0.01 | 0.0002 | 0.0002 | 0.0002 | 0.0002 | 0.0002 | 0.0002 | 0.0002 | 0.0002 |
| 0.03 | 0.0007 | 0.0007 | 0.0007 | 0.0007 | 0.0007 | 0.0007 | 0.0007 | 0.0006 |
| 0.05 | 0.0011 | 0.0011 | 0.0011 | 0.0012 | 0.0012 | 0.0012 | 0.0011 | 0.0011 |
| 0.1 | 0.0022 | 0.0021 | 0.0022 | 0.0024 | 0.0023 | 0.0024 | 0.0022 | 0.0021 |
| 0.2 | 0.0047 | 0.0047 | 0.0046 | 0.0047 | 0.0047 | 0.0046 | 0.0044 | 0.0041 |
| 0.4 | 0.0084 | 0.0084 | 0.0085 | 0.0090 | 0.0087 | 0.0083 | 0.0083 | 0.0078 |
| 0.5 | 0.0110 | 0.0112 | 0.0112 | 0.0109 | 0.0112 | 0.0110 | 0.0108 | 0.0104 |
