# Supplementary material for: An open-source probabilistic record linkage process for records with family-level information: Simulation study and applied analysis
Source: PLoS One. 2023 Oct 20;18(10):e0291581. doi: 10.1371/journal.pone.0291581 (PMC10588881; doi:10.1371/journal.pone.0291581)
Supplement: S4 Table — (DOCX) [file pone.0291581.s009.docx]

| Table S4. Overlap of LinkPlus and ChoiceMaker Matched pairs with varied thresholds for ChoiceMaker. | | | | | | |
| --- | --- | --- | --- | --- | --- | --- |
| Threshold | Total Pairs Matched by ChoiceMaker | True Pairs Matched by ChoiceMaker | Pairs Matched by ChoiceMaker Only | Pairs Matched by LinkPlus Only | % True ChoiceMaker Pairs in All Pairs Matched by ChoiceMaker | % True ChoiceMaker Pairs in All Pairs Matched by LinkPlus |
| 0.8 | 59,685 | 56,732 | 2,953 | 1,645 | 95.05% | 97.18% |
| 0.85 | 59,619 | 56,695 | 2,924 | 1,682 | 95.10% | 97.12% |
| 0.9 | 59,556 | 56,661 | 2,895 | 1,716 | 95.14% | 97.06% |
| 0.95 | 59,316 | 56,501 | 2,815 | 1,876 | 95.25% | 96.79% |
| 0.99 | 58,670 | 56,174 | 2,496 | 2,203 | 95.75% | 96.23% |
